# 

**Published:** 1889-01-05

**Authors:** 


### THE HOSPITAL

A WEEKLY INSTITUTIONAL JOURNAL OF
**Science, Medicine, Nursing, and Philanthropy.**

### CONTENTS.

**Is a Register for Nurses Desirable?** - 211

**Royal Albert Orphan Asylum.** By R. WITHERBY, Secretary 212

**The Doctor's Pulpit.**—"All Sorts and Conditions" of Women.—I. - 213

**Metropolitan Hospitals Ancient and Modern.**—II. - 214

**Editor's Letter Box.**—Jane and Eliza Wigham - 215

**Within the Wards.**—"Alcoholic" Diseases—"Tinned" Meats—Brain Injury and Bad Temper - 216

**Babies.** I.—The World's Babies - 217

**Notes and News** - 218

**Everybody's Page** - 220

**Annotations.**—The Sweating of Nurses—The Eating-house Trick—Suicide and Insanity - 221

**Diphtheria** - 222

**False Impressions.** By CAROLINE FOTHERGILL, Author of "An Enthusiast," "A Voice in the Wilderness," etc. - 223

**The Students' Column.**—University of London - 224

**In and Out Among the Hospitals.** By a SECRETARY OF TEN YEARS' STANDING - 225

**Scraps and Gleanings.—Amusements and Relaxation** 226

**EXTRA SUPPLEMENT—THE NURSING MIRROR.**

**En Passant.**—Bristol Nurses' Home—Christmas Festivities—Pity the Poor Pro.—Wages and Washing—A Disorganised Infirmary—Bedford Wants Nurses - liii

**Is a Register for Nurses Desirable?** By a DOCTOR - liv

**Lectures to Nurses** - lv

**Women as Future Druggists.—Private Nursing** - lv

**Everybody's Opinion.**—The Pension Fund—Wages and Washing—Philanthropic Sweating - lvi

**Presentations.—Appointments** - lvi

**Vacancies.—Notes and Queries** - lvi

